# Editorial: Images from red cells, Volume II

**DOI:** 10.3389/fphys.2023.1252273

**Published:** 2023-07-26

**Authors:** Giampaolo Minetti, Paola Bianchi, Anna Bogdanova, Lars Kaestner

**Affiliations:** ^1^ Department of Biology and Biotechnology “L. Spallanzani”, Laboratories of Biochemistry, University of Pavia, Pavia, Italy; ^2^ Hematology Unit, Physiopathology of Anemias Unit, Fondazione IRCCS Ca’ Granda Ospedale Maggiore Policlinico Milano, Milan, Italy; ^3^ Red Blood Cell Research Group, Institute of Veterinary Physiology, and Center for Clinical Studies (ZKS), Vetsuisse Faculty, University of Zurich, Zurich, Switzerland; ^4^ Zurich Center for Integrative Human Physiology (ZIHP), Zurich, Switzerland; ^5^ Theoretical Medicine and Biosciences, Medical Faculty, Saarland University, Homburg, Germany; ^6^ Dynamics of Fluids, Experimental Physics, Saarland University, Saarbrücken, Germany

**Keywords:** red blood cell morphology, microscopy, anemia, RBC membrane defects, cytoskeleton

Seeing is believing. In this respect, “*Images from red cells*” is a compelling concept. In analogy, also seeing each other as protagonists of the red blood cell (RBC) community (European Red Cell Society, ERCS), not only virtually as forced by the COVID-19 pandemic (Bianchi et al., 2023), but in person during the (under normal conditions organized every 2 years) meeting of the ERCS 2022 at *Villa Cagnola* near Varese, Italy, was a convincing action. On that occasion, approximately 100 researchers from 14 different countries came together. The topics included normal and pathological erythropoiesis, clinical hematology, red blood cells (RBCs) as carriers, cultured RBCs, artificial blood, and RBC membrane structure and functions as well as new technologies in the study of RBCs. Some of the contributions conceived at the meeting have been collected in this Research Topic.

Entering into details, we ordered the contributions according to the spatial resolution of the imaging technique applied or referred to—starting with the lowest resolution still providing sufficient information about the red cell phenotype. Increasing resolution down to a few nanometers allows the investigation of molecular structures.

From the finest characterization (i.e., pathogenic variants identified by targeted NGS) to the biochemical defect and to red cell morphology. This is the approach used by Vercellati et al. who examined the genotypes of 25 patients affected by hereditary spherocytosis (mutations in *EBP42*, *SLC4A1*, *SPTA1*, *SPTB*, and *ANK1*) and related them to the biochemical defect observed by SDS-PAGE electrophoresis (Bianchi et al., 2020; Fermo et al., 2021). So, the imaging is not the imaging of cells but the imaging of processed cell extracts. Surprisingly, a direct correspondence between the biochemical lesion and the molecular defect was identified only in approximately half cases (11/25), mostly with band 3 deficiency due to *SLC4A1* mutations. Most of the mutations in *SPTB* and *ANK1* genes did not result in abnormalities of RBC membrane protein; conversely, in two cases, the molecular lesion did not correspond to the biochemical defect, suggesting that a mutation in a specific membrane-skeletal protein may result in more complex RBC membrane damage or suffering.

Danusso et al. used confocal microscopy and partly 3D volume rendering to investigate the effect of fixatives and anticoagulants on human umbilical cord blood cells. Although the study does not provide new insights into the biology of cord blood cells, this methodological investigation is important when using experimental quantitative data of cell size for computational modeling of physiological and pathophysiological processes. Except for monocytes (citrate), anticoagulants had no effect on the cell size, while preservatives showed a concentration-dependent shrinkage of the cells similar to that shown before for circulating red blood cells (Abay et al., 2019).

Bernecker et al. used a combination of optical tweezers, atomic force microscopy, and digital holographic microscopy to approach a hot frontier topic in red cell physiology, the culturing of RBCs for transfusion purposes. Continued technical development has allowed optimization of culturing conditions to afford ever-increasing yields of enucleated reticulocytes and production scaling up. However, a major, still unsolved issue is the obtainment of fully mature RBCs, characterized by mechanical stability, which is in turn linked to the complete assembly of the membrane skeleton and its anchoring to the lipid bilayer. By a combination of advanced microscopy techniques, Bernecker et al. observed changes in the biomechanical properties of human reticulocytes cultured *in vitro* from CD34^+^ hematopoietic stem/progenitor cells isolated from peripheral blood of adult donors. They performed the culture under two different conditions in a medium of normal lipid composition or in a lipid-depleted medium and compared the properties of the cultured reticulocytes with those of normal reticulocytes from cord blood. Results show that reticulocytes produced in the normal medium display biomechanical properties similar to those of native cord blood reticulocytes. Conversely, culturing of erythroid precursors in the lipid-deficient medium leads to the production of reticulocytes of spherical shape, abnormal membrane composition, and severely impaired biomechanical functions, highlighting the often-neglected role that lipids play not only during erythroid development but also in the terminal maturation of circulating reticulocytes (Minetti et al., 2023).

From atomic force microscopy to optical morphology using artificial intelligence (AI). With the advent of AI, which enables us to analyze and elaborate an impressive number of data at the same time, the possibility to in deep investigate RBC morphology came to reality (Kaestner, 2020). This is the case of a new tool to investigate RBC morphology by the support of AI. Sadafi et al. introduced in their contribution an AI tool for the interpretable analysis of RBC morphology, RedTell. The authors demonstrated RedTell’s applicability and power under different conditions: to distinguish patients suffering from different diseases, to the classification of cells into echinocytes, discocytes, and stomatocytes, and to distinguish sickle cells in sickle cell disease patients.

Finally, there is a paper in this Research Topic, which does not use any classical imaging method but laser/fluorescence-based flow cytometry and rotational thromboelastometry to characterize molecular and functional properties of stored packed red blood cells. Öhlinger et al. showed that clotting times and clot formation times were considerably increased in samples reconstituted with RBCs from stored packed samples compared to fresh red blood cells. From this study one can conclude: 1) there is no evidence for an amplified clotting process from prolonged storage of packed red blood cells as long as it refers to parameters that can be assessed by rotational thromboelastometry. 2) The quality control of stored RBCs is an urgent and demanding task that can be tackled by various methods (Regulation of the European Parliament, 2002; Lopes et al., 2023) and should include coagulation properties. 3) The study adds to accumulating evidence viewing red blood cells as active players in the clotting process (Bernhardt et al., 2019).

In summary, the contributions presented in this Research Topic underline and reinforce the interest in the fascinating RBCs and raise the expectations of the contributions that will be presented in the next ERCS meeting, which is scheduled for 2024 in the Netherlands.
